# Regulatory T Cells in Endemic Burkitt Lymphoma Patients Are Associated with Poor Outcomes: A Prospective, Longitudinal Study

**DOI:** 10.1371/journal.pone.0167841

**Published:** 2016-12-29

**Authors:** Emily Parsons, Juliana A. Otieno, John Michael Ong’echa, Christina E. Nixon, John Vulule, Christian Münz, V. Ann Stewart, Ann M. Moormann

**Affiliations:** 1 Uniformed Services University of the Health Sciences, Bethesda, MD United States of America; 2 Jaramogi Oginga Odinga Teaching and Referral Hospital, Ministry of Health, Kisumu, Kenya; 3 Center for Global Health Research, Kenya Medical Research Institute, Kisumu, Kenya; 4 Center for International Health and the Department of Pathology, Rhode Island Hospital, Providence, RI United States of America; 5 Department of Viral Immunobiology, Institute of Experimental Immunology, University of Zürich, Zürich, Switzerland; 6 Program in Molecular Medicine, University of Massachusetts Medical School, Worcester, MA United States of America; University of North Carolina at Chapel Hill, UNITED STATES

## Abstract

Deficiencies in Epstein-Barr virus (EBV)-specific T cell immunosurveillance appear to precede the development of endemic Burkitt lymphoma (eBL), a malaria-associated pediatric cancer common in sub-Saharan Africa. However, T cell contributions to eBL disease progression and survival have not been characterized. Our objective was to investigate regulatory and inflammatory T cell responses in eBL patients associated with clinical outcomes. By multi-parameter flow cytometry, we examined peripheral blood mononuclear cells from 38 eBL patients enrolled in a prospective cohort study in Kisumu, Kenya from 2008–2010, and 14 healthy age-matched Kenyan controls. Children diagnosed with eBL were prospectively followed and outcomes categorized as 2-year event-free survivors, cases of relapses, or those who died. At the time of diagnosis, eBL children with higher CD25^+^Foxp3^+^ regulatory T (Treg) cell frequencies were less likely to survive than patients with lower Treg frequencies (p = 0·0194). Non-survivors also had higher absolute counts of CD45RA^+^Foxp3^lo^ naïve and CD45RA^-^Foxp3^hi^ effector Treg subsets compared to survivors and healthy controls. Once patients went into clinical remission, Treg frequencies remained low in event-free survivors. Patients who relapsed, however, showed elevated Treg frequencies months prior to their adverse event. Neither concurrent peripheral blood EBV load nor malaria infection could explain higher Treg cell frequencies. CD8^+^ T cell PD-1 expression was elevated in all eBL patients at time of diagnosis, but relapse patients tended to have persistently high PD-1 expression compared to long-term survivors. Non-survivors produced more CD4^+^ T-cell IL-10 in response to both Epstein-Barr Nuclear Antigen-1 (EBNA-1) (p = 0·026) and the malaria antigen *Plasmodium falciparum* Schizont Egress Antigen-1 (p = 0·0158) compared to survivors, and were concurrently deficient in (EBNA-1)-specific CD8^+^ T-cell derived IFN-γ production (p = 0·002). In addition, we identified the presence of Foxp3^-^IL10^+^ regulatory Type 1 cells responding to EBNA-1 in contrast to the malaria antigen tested. These novel findings suggest that poor outcomes in eBL patients are associated with a predominantly immuno-regulatory environment. Therefore, Treg frequencies could be a predictive biomarker of disease progression and manipulation of Treg activity has potential as a therapeutic target to improve eBL survival.

## Introduction

Endemic Burkitt lymphoma (eBL) is an aggressive monoclonal B cell lymphoma and one of the most common pediatric cancers in Equatorial Africa [1, 2]. Tumors are associated with Epstein-Barr virus (EBV) [3], a ubiquitous gamma herpes virus that establishes life-long latency in resting B cells and is predominantly controlled by a T cell mediated immune response. Primary EBV infection in sub-Saharan Africa occurs during infancy, so that by three years of age almost 100% of children are EBV sero-positive [4]. In addition to EBV, co-infection with *Plasmodium falciparum* (Pf) malaria has been linked to eBL pathogenesis, and studies have shown that malaria can induce polyclonal B cell expansion and impair EBV-specific T cell immunity [5, 6]. However, there is little knowledge of the role T cell immunity plays in eBL disease progression and long-term survival.

In addition to T cell pro-inflammatory responses, EBV induces a regulatory response that includes the induction of IL-10 and the presence of EBV-specific regulatory T (Treg) cells [7, 8]. The balance between EBV-specific inflammation and regulation is important for viral control with limited immunopathology. Infectious mononucleosis, caused by primary EBV infection in adults and adolescents, is associated with an abundance of EBV-specific pro-inflammatory responses, with symptom resolution upon an expansion of regulatory responses [9]. Although eBL tumor cells display latency I characterized by the sole expression of the EBV latent antigen Epstein-Barr Nuclear Antigen-1 (EBNA-1) [10], anti-viral immune responses to EBNA-1 appear insufficient for tumor control. This characteristic has been observed in other EBV-infected tumors and may be related to T cell suppression [11–13].

Higher levels of Foxp3^+^ regulatory T (Treg) cells have been reported in numerous cancers [14], including other EBV-associated tumors [15], and are thought to limit anti-tumor immunity. However, not all reports have found a correlation between high Treg levels and poor outcomes [16–18]. The goal of this study was to investigate the regulatory T cell populations and their predictive value for disease outcome in children diagnosed with eBL.

Using a longitudinal cohort of eBL patients in western Kenya, we tested the hypothesis that patients with poor outcomes have higher regulatory responses against EBV, and that low frequencies of Treg cells is associated with long-term survival.

## Materials and Methods

### Study participants

The demographic characteristics and chemotherapeutic treatment regimen of our eBL study population from western Kenya has been described in more detail elsewhere [19]. For this sub-study, we included 38 eBL patients between the ages of 2–13 years old (median age 6.7 years), who were prospectively enrolled between August 2008 and January 2010 upon admittance to Jaramogi Oginga Odinga Teaching and Referral Hospital (JOOTRH) in Kisumu, Kenya. Diagnosis was confirmed by fine needle aspiration cytopathology. Patients were excluded if eBL diagnosis could not be confirmed by cytopathology, if they were HIV positive, if the chemotherapy regimen did not follow the hospital’s standard protocol, if parents withdrew their child from the study, or if the outcome was not known. Blood samples were taken at admission prior to commencement of chemotherapy, at the end of their in-patient treatment, and at monthly follow-up time points as the patients returned to the hospital to receive maintenance out-patient therapy. Age-matched healthy controls (median age 6.5 years, range 2.7–13.0) were from Kisumu District, a region of malaria holoendemnicity. Healthy control children were not infected with malaria at the time their blood was drawn even though they were residents of the same malaria holoendemic region.

Ethical approval for this study was obtained from the Ethical Review Committee at the Kenya Medical Research Institute, JOOTRH, and from the Institutional Review Board at the University of Massachusetts Medical School (UMMS). Legal guardians of study participants gave written informed consent. Children over the age of 7 years gave assent.

### Quantification of blood stage malaria infection and EBV DNA

DNA was isolated from peripheral blood cells using Qiagen DNAeasy kit (Qiagen) for Pf malaria and EBV. Quantification of EBV load by polymerase chain reaction has been described elsewhere [20]. Briefly, primers and probes amplified a 70-basepair region of the EBV BALF5 gene, and the Pf 18s rRNA gene [21].

### Cell culture and stimulation

PBMCs were isolated by Ficoll-Hypaque density gradient centrifugation, cryopreserved and transferred to UMMS for further studies. After thawing, PBMCs were cultured in RPMI with 10% heat inactivated fetal bovine serum, supplemented with L-glutamine (2mM), penicillin (100 IU/mL), and streptomycin (100 μg/mL). After resting overnight, cells were stimulated with either an EBNA-1 peptide pool (10 μg/mL) or with PfSEA-1 antigen (10 μg/mL) for 16–18 hours in the presence of anti-CD49d and anti-CD28 [22, 23]. Negative (PBS containing equivalent volume of the EBNA-1 peptide carrier, DMSO) and positive (staphylococcus enterotoxin B, 10 μg/mL [ToxinTechnologies]) controls were included for each patient sample. GolgiPlug (BD) was added after 2 hours of stimulation.

### Flow cytometry

Cells were washed and stained with Live/dead fixable violet dead cell stain kit (Life Technologies). After additional washing, cells were stained with surface markers for 15 minutes at room temperature. The Foxp3 Staining Buffer Set (eBioscience) was used to fix and permeabilize cells prior to staining for intracellular markers. The following antibodies were used: CD3-A700 (SP34-2), CD4-BV605 (RPA-T4), CD8-BUV395 (RPA-T8), CD19-BV421 (H1319), IFN-γ-FITC (4s.B3), CCR7-BV786 (3D12), IL-10-PE-TR (JES3-19F1), CD45RA-APC-H7 (H100), CD14-BV421 (MΦP9) (all from BD Biosciences), CD25-APC (BC96, eBioscience), Foxp3-PE (206D, BioLegend), PD-1-PE-Cy7 (EH12.2H7, BioLegend). Fluorescence Minus One controls were used to establish gates (S1 Fig). Flow cytometry was performed on an LSR II flow cytometer (BD Biosciences) and data were analyzed on FlowJo X.0.6 (Tree Star).

### Data analysis

Complete blood count (CBC) was obtained at each venous blood sampling and used to convert T cell subset frequencies obtained by flow cytometry to absolute cell counts per μL by assuming that the proportion of CD3^+^ T cells found in lymphocytes identified by forward scatter and side scatter in flow cytometry would equal that found in lymphocytes identified by CBC. Lymphocyte counts obtained from study participants can be viewed in S2 Fig.

To assess for analysis of covariance with participant age, we transformed dependent values to a log scale and used SPSS version 22 for Windows. GraphPad Prism version 6.00 for Windows (GraphPad Software) was used for all other analyses. Kruskal-Wallis or Mann-Whitney Test were used where appropriate to compare between groups. Friedman Test or Wilcoxon Matched-Paris Signed Rank test were used for paired samples. We classified a cytokine (IFN-γ or IL-10) response as positive if it was greater than 2-fold above the unstimulated control. Fisher’s Exact Test was used to compare frequency of responders. A chi-squared test was used to compare IL-10^+^ cell types between IL-10 responders to EBNA-1 versus PfSEA-1. To correlate Treg frequencies with EBNA-1-specific cytokine responses, both values were transformed to a log scale. Treg frequencies with values of 0 were assigned a frequency half that of the lowest detected frequency (0.00376) to allow geometric statistical comparisons.

## Results

### Participant characterization

We evaluated peripheral blood mononuclear cells (PBMCs) from a total of 38 eBL patients: 21 who did not survive their in-patient induction phase chemotherapy and died within 6 weeks of diagnosis (non-survivors), 14 patients who became event-free survivors, and 3 patients who relapsed less than 2 years after discharge. Non-survivors included in this study tended to be younger (median age 5.3 years, range 2.3–9.0) than survivors (median age 9.0 years, range 3.8–13.4), relapse patients (median age 6.7, range 5.8–10.4), and healthy controls (median age 6.5 years, range 2.7–13.0 years). In analysis of covariance, age did not exert a significant effect, and in the overall study population age is not a predictor of survival [19]. Complete blood count was used to convert cell frequencies into absolute counts; N = 14 non-survivors, 11 survivors, 11 healthy controls.

### Regulatory T cell frequencies are lower in survivors

**Fig 1:**
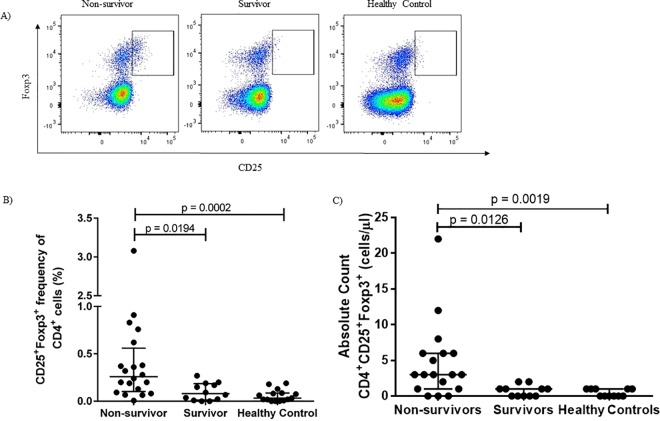
CD25^+^Foxp3^+^ Treg cells are associated with poor outcome. Identification of CD25^+^Foxp3^+^ Treg cells by flow cytometry (A). At the time of diagnosis, frequencies and (B) absolute cell counts (C) of CD25^+^Foxp3^+^ cells are higher in eBL patients who died than in survivors and healthy controls.

At the time of eBL diagnosis and prior to induction of chemotherapy, frequencies of CD4^+^CD25^+^Foxp3^+^ cells were significantly higher in non-survivors than in survivors and healthy controls (Fig 1A and 1B). These patterns held true when frequencies of CD25^+^Foxp3^+^ cells were converted into absolute cell numbers (Fig 1C).

**Fig 2:**
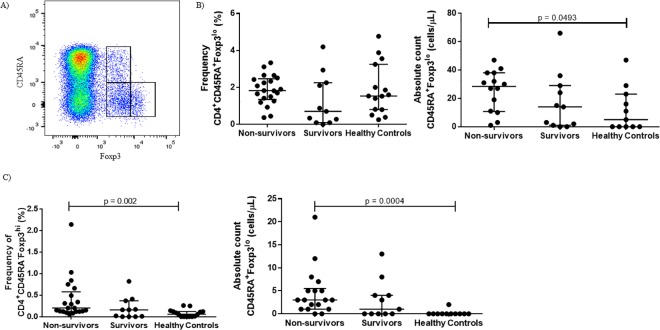
Higher numbers of both CD45RA^+^Foxp3^lo^ naïve Treg (nTreg cells) and CD45RA^-^Foxp3^hi^ effector Treg (eTreg) cells are associated with poor outcome. (A) Gating strategy differentiating nTreg and eTreg cells from CD45RA^-^Foxp3^lo^ non-Treg cells. At the time of diagnosis, eBL non-survivors have higher absolute cell counts of both nTreg (B) and eTreg (C) cell subtypes compared to eBL survivors and healthy controls.

Some of the discord in whether or not Treg cells in cancer are associated with poor outcome has been attributed to the pitfalls of relying on CD25 and Foxp3 for Treg cell identification, given that effector T (Teff) cells transiently express both markers upon activation [24, 25]. Miyara *et al*. developed a method to avoid inclusion of activated Teff cells in the Treg gate by using CD45RA expression to distinguish Treg cells from activated CD45RA^-^Foxp3^lo^ non-Treg cells [26]. Naïve Treg (nTreg) cells are CD45RA^+^Foxp3^lo^, and effector Treg (eTreg) cells are CD45RA^-^Foxp3^hi^ [26]. Using this gating strategy (Fig 2A), we found although there was no difference in the frequency of nTreg cells between the groups, there was a higher frequency of eTreg cells in eBL non-survivors compared healthy controls and eBL survivors (Fig 2B and 2C). When these frequencies were converted into absolute cell counts, non-survivors had higher numbers of both nTreg and eTreg cells compared to healthy controls with intermediate-low median levels for the eBL survivors. The nTreg and eTreg gating strategy resulted in higher absolute numbers of Treg cells compared to gating on CD25^+^Foxp3^+^, perhaps because we maintained a strict gating strategy for the identification of CD25^+^ cells, but the overall relationships remain the same with either gating strategy.

**Fig 3:**
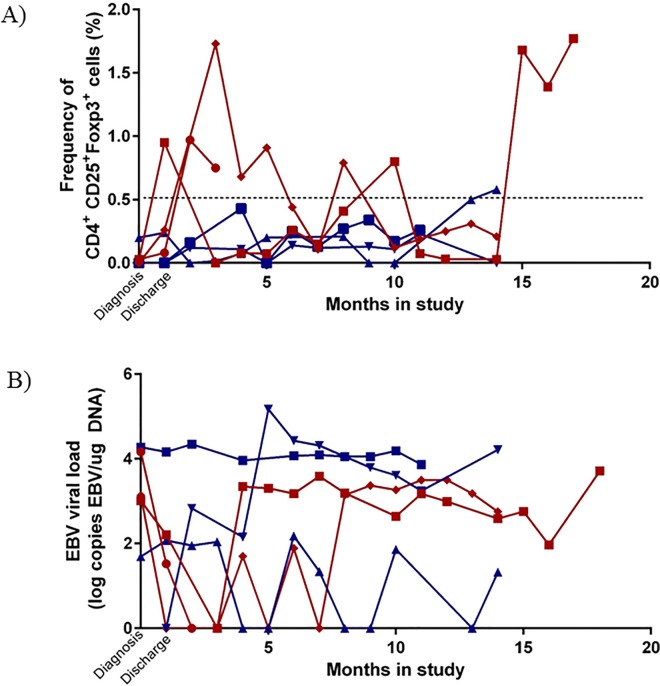
Elevated Treg frequencies in patients prior to relapse. Frequencies of CD25^+^Foxp3^+^ Treg cells (A) and EBV viral load (B) at monthly follow-up time points in 3 event-free survivors and 3 patients who relapsed. Proposed Treg frequency threshold indicative of future relapse was established by measuring Treg frequencies in 8 other event-free survivors 4–9 months post discharge, and frequencies never rose above 0.5% (dotted line). Designated as a red ♦ = BL001 (relapse), designated as a red ● = BL002 (relapse), designated as a red ■ = BL003 (relapse), designated as a blue ▲ = BL004 (event-free survivor), designated as a blue ▼ = BL005 (event-free survivor) designated as a blue ■ = BL006 (event-free survivor).

To determine longitudinal changes in Treg populations over time, we measured at each available monthly interval the Treg frequencies of three survivors and three patients who relapsed (range of 4–14 time points per person over a maximum of 18 months). Though all 6 patients had relatively low Treg frequencies at diagnosis, long-term survivors’ Treg frequencies remained low during remission compared to peaks of elevated frequencies in patients who relapsed (Fig 3A). Interestingly, relapse occurred between 12 months (patient BL002) and < 1 month (patient BL003) after the latest time points we were able to analyze, yet their Treg frequencies began to peak in some cases 12 months prior to relapse. To confirm that Treg frequencies remain low in long-term survivors, we examined additional follow-up time points 4 to 9 months after discharge in 8 other patients. Treg frequencies during remission remained low; at every time point examined, the CD25^+^Foxp3^+^ frequency did not exceed 0.5%. EBV was intermittently detectable at appreciable viral loads in peripheral blood samples during follow-up however there was no viral pattern predictive of relapse or survival (Fig 3B).

### Regulatory T cell frequencies do not correlate with malaria parasitemia or EBV load

We examined the relationships between the frequency of Treg cells and concurrent infections with two diseases important in eBL pathogenesis, EBV and malaria, in order to determine if they contributed to Treg expansion. All study participants, including healthy controls, resided in malaria holoendemic regions, so while we could not directly measure previous malaria exposure, we presume long-term exposure was similarly high between groups. When we stratified by whether an eBL patient had a current malaria blood stage infection (N = 8) at time of hospital admission, we saw no difference in Treg frequency or numbers in any Treg subset. There was also no correlation between current malaria blood stage infection and IFN-γ or IL-10 production upon stimulation with the malaria antigen PfSEA-1 (S3 Fig).

EBV load has not been identified as a stand-alone biomarker for eBL, as healthy children residing in areas of high malaria transmission harbor relatively high EBV titers [27] yet fail to progress to eBL. However, we wanted to examine the influence of high viral loads as a possible effect modifier on Treg frequencies and cytokine levels. We found no correlation between peripheral blood EBV load and Treg cell numbers, or any correlation between IFN-γ or IL-10 production and viral load. We also stratified eBL patients by St. Jude/Murphy tumor staging and found no association with Treg frequencies (S3 Fig) [28] suggesting that Treg cells may play a role within tumor stage classification in influencing outcome.

### T cells in eBL patients have higher PD-1 expression

**Fig 4:**
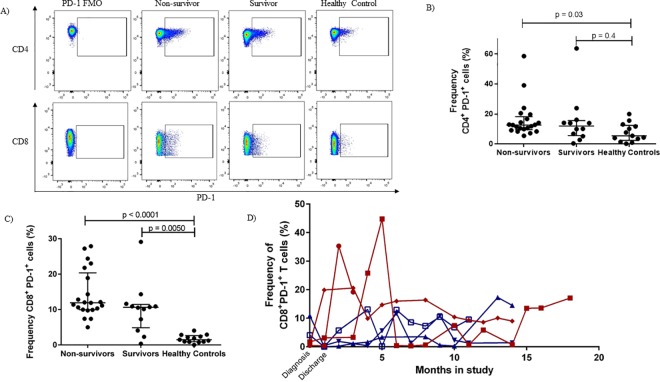
T cells in eBL patients have higher PD-1 expression than in healthy controls. Gating strategy for the identification of PD-1^+^ cells (A). Frequency of CD4^+^ and CD8^+^PD-1^+^ cells at the time of diagnosis in eBL non-survivors, eBL survivors and healthy controls (B and C, respectively). In general PD1 expression on CD4 and CD8 T cells was higher in eBL non-survivors compared to age-matched malaria exposed yet health controls. eBL survivors had intermediate levels of PD1 expressed on their CD4 T cells yet levels as high as non-survivors for their CD8 T cells. Patients who relapsed tended to have peaks of elevated CD8^+^PD-1^+^ frequencies over time (D). Designated with a red ♦ = BL001 (relapse), designated with a red ● = BL002 (relapse), designated with a red ■ = BL003 (relapse), designated with a blue ▲ = BL004 (event-free survivor), designated with a blue ▼ = BL005 (event-free survivor) designated with a blue ■ = BL006 (event-free survivor). FMO; Fluorescence Minus One.

Children living in malaria holoendemic areas seem to have EBV-specific CD8^+^ T cells with a more exhausted phenotype compared to age-matched children residing in an area with little to no malaria transmission [29]. In this study that includes children with eBL, who have been residing in malaria endemic areas, we found that overall CD4^+^ PD-1 expression was significantly elevated in non-survivors (Fig 4B) while all eBL patients had higher expression of PD-1 on CD8^+^ T cells compared to healthy malaria-exposed controls (Fig 4C). Similar to the longitudinal Treg frequencies, eBL patients who relapsed had periods of elevated CD8^+^PD-1^+^ T cell frequencies (Fig 4D) compared to long-term survivors.

Using CD45RA and CCR7 markers to differentiate between effector memory T cells (T_EM_), central memory T cells (T_CM_), RA^+^ effector memory T cells (T_EMRA_) or naïve T cells (T_N_) [30], there was no difference in proportion of these CD4 and CD8 T cell subsets between eBL patients and healthy controls, or between patients who died and patients who survived (S4 Fig). This suggests that non-survival is not due to a global shift in T cell lineage that would alter the development of central or effector memory subtypes but is associated with PD-1 expression and expansion of specific regulatory subsets.

### EBNA1-specific T cell responses differ by outcome

**Fig 5:**
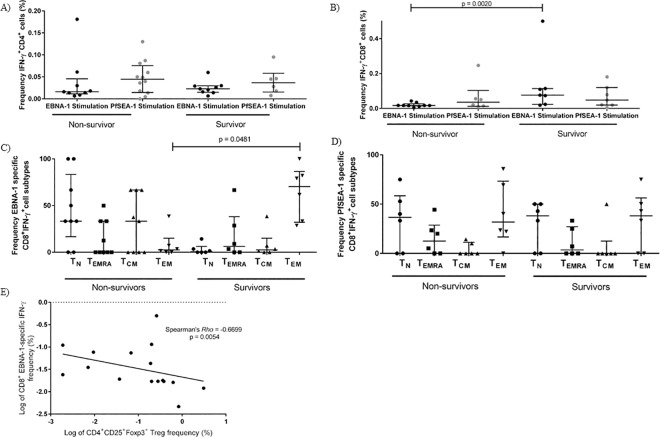
Non-survivors produce less EBV-specific IFN-γ, and from difference T cell subsets. Among IFN-γ producers, there was no difference in the magnitude of CD4^+^ T cell IFN-γ production in response to EBNA-1 or PfSEA-1 stimulation between eBL survivor and non survivors (A), non-survivors produced less CD8^+^ T cell IFN-γ from EBNA-1 stimulated cells (B). Among EBNA-1-specific CD8^+^ T cell IFN-γ producers, survivors produced more CD8^+^ T cell IFN-γ from the CD45RA^-^CCR7^-^ effector memory (T_EM_) cell subset than non-survivors (C). Among PfSEA-1-specific CD8^+^ T cell IFN-γ producers, there was no difference between survivors and non-survivors in the cell type that produced IFN-γ (D). Among IFN-γ T cell responders in both survivors and non-survivors, CD25^+^Foxp3^+^ Treg frequency negatively correlated with CD8^+^ T cell EBNA-1 IFN-γ production (E).

T cell responses are important for limiting pathology during EBV persistence and reactivation [31]. Previous work on a similar eBL cohort using ELISPOTs showed that eBL patients were deficient in IFN-γ responses to EBNA-1 compared to robust responses in healthy controls [22]. Using flow cytometry, we found no difference in the frequency of IFN-γ T cell responses to EBNA-1 comparing all eBL patients to healthy age-matched controls within either CD4^+^ T cells (p = 0·2591, Fisher’s exact test) or CD8^+^ T cells (p = 0·2719, Fisher’s exact test) (S1 Table). However, when eBL children were stratified by outcome, EBNA-1-specific IFN-γ produced by CD4+ T cells did not differ but the CD8^+^ T cell responses were significantly lower in non-survivors compared to survivors (p = 0·0200, Mann-Whitney test) (Fig 5A and 5B). In addition, when comparing T cell subset proportions in response to EBNA-1-specific stimulation, survivors produced IFN-γ^+^ predominantly from the CD8^+^ CD45RA^-^CCR7^-^ T_EM_ subset (Fig 5C) in contrast to non-survivor who had more IFN-γ responses derived from the CD45RA^+^CCR7^+^, the naïve-like T cell subset. There were no differences in the PfSEA-1-specifc CD8^+^IFN-γ^+^ T cell subsets between eBL survivors and non-survivors, as expected (Fig 5D), confirming that eBL patients have a selective immune-deficiency to EBNA-1. Moreover, there was a negative correlation between EBNA1-specific CD8^+^ T cell IFN-γ^+^ and CD4^+^ Treg cell frequencies (Fig 5E) further demonstrating an immune-regulatory imbalance.

**Fig 6:**
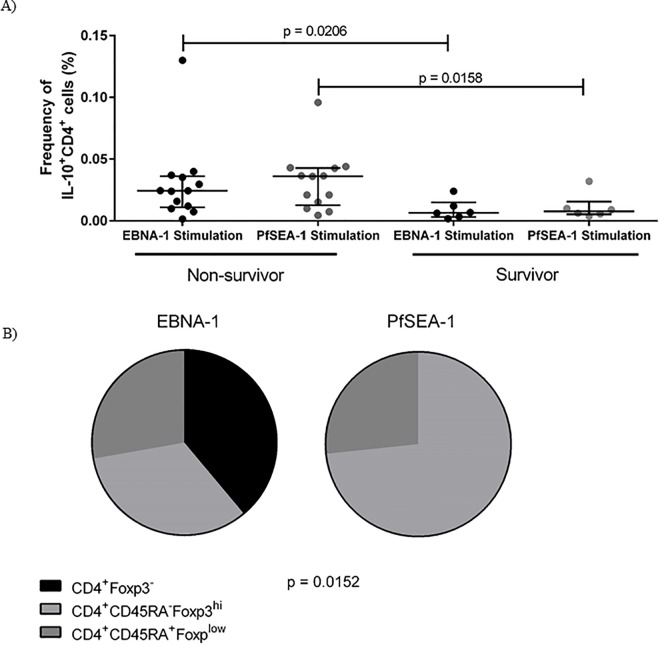
Non-survivors produce more IL-10 than survivors. A) Comparing only patients who produced IL-10 above background upon stimulation, the magnitude of CD4^+^ T cell IL-10 response was greater in eBL non-survivors when stimulated with either EBNA-1 peptide pool or PfSEA-1-1 peptides. B) Among non-survivors, some IL-10 was from different Treg sources, including CD45RA^+^Foxp3^lo^ naïve Treg (nTreg) and CD45RA^-^Foxp3^hi^ effector Treg (eTreg), but was also produced from Foxp3^-^ CD4^+^ T cells. Pie charts represent numbers of patients who produced IL-10 from one of the three sources of CD4^+^ T cell comparing EBNA1 and PfSEA1.

The overall frequency of CD4^+^ T cell IL-10 responders was significantly higher among eBL patients than healthy controls for both EBNA-1 (p = 0·0124, Fisher’s exact test) and PfSEA-1 (p = 0·0068, Fisher’s exact test). Moreover, among eBL patients, non-survivors produced a higher magnitude of IL-10 when stimulated both with EBNA-1 and PfSEA-1 (Fig 6A). Cellular sources of IL-10 were found to be heterogeneous; arising within regulatory nTreg and eTreg subpopulations. However, this did not account for all IL-10 production. IL-10 in response to EBNA1 was also produced within the CD4^+^ T cell Foxp3^-^ populations in contrast to the malaria antigen, PfSEA-1 that only stimulated IL-10 from Foxp3^+^ Tregs (Fig 6B).

## Discussion

Due to the challenges of conducting clinical studies of pediatric patients residing in sub-Saharan Africa, knowledge of the cellular immune response to eBL lags behind that of many cancers in developed countries. In this study, we found that Foxp3^+^ Treg cells are associated with poor outcomes in children with eBL. At the time of diagnosis, non-survivors had higher frequencies of CD25^+^Foxp3^+^ Treg cells compared to survivors and healthy controls, a pattern confirmed with the more recently described CD45RA^+^Foxp3^lo^ nTreg and CD45RA^-^Foxp3^hi^ eTreg cells [26]. Our study found that absolute cell counts of nTreg cells and eTreg cells were significantly higher in non-survivors compared to survivors and healthy controls. There was no statistical difference in the absolute lymphocyte count between the groups, although we noted several children with apparent lymphopenia, paradoxically in eBL survivors prior to commencement of chemotherapy. Acute malaria has been associated with transient lymphopenia but lymphocyte counts did not correlate with active malaria infections within our study population. Future studies will explore causes of lymphopenia within our eBL cohort and correlate peripheral lymphocyte count with magnitude of tumor infiltrating lymphocytes.

There was no association between Treg frequency and tumor stage, concurrent malaria infection or peripheral blood EBV viral load, suggesting differences in Treg frequencies reflect intrinsic differences in human immune regulation. Longitudinally, Treg frequencies in event-free eBL survivors fluctuated within a limited range, generally < 0.5%. In contrast, Treg frequencies fluctuated dramatically in patients who relapsed, with elevated peaks above the ‘event-free threshold’ even months preceding relapse. Future work would seek to clarify whether high Treg numbers cause this lack of tumor control, whether Treg numbers increase in response to tumor-related inflammation, or whether they are induced by additional exposure to the EBV antigen EBNA-1 as tumors become refractory to chemotherapy. For this study we were unable to biopsy tumors for assessment of the immunological microenvironment and so cannot confirm whether differences in Treg frequencies associated with poor outcomes also occur within the tumor infiltrating lymphocytes. However, measuring more easily accessible Treg cell frequencies that are detectable in the peripheral blood compartment could provide a predictive biomarker to identify patients at risk of relapse.

Our previous study found that markers of EBV-specific CD8^+^ T cell exhaustion were higher in children residing in malaria holoendemic areas compared to expression in children who were not exposed to many if any malaria infections, and this was specific to T cell responses to EBV [29]. Along this line of inquiry, we found that CD8^+^ T cell PD-1 levels were even higher in children with eBL compared to healthy controls matched for cumulative malaria exposure. The development of T cell exhaustion goes hand in hand with the development of tolerance; both arise after prolonged exposure to an antigenic stimulus and are associated with a diminished antigen-specific inflammatory response. Although we did not find differences in PD-1 expression between eBL non-survivors and survivors, we did find differences between eBL patients and healthy children. Future studies will explore the relationship between EBV-specific T cell exhaustion and the development of Treg cells within eBL patients and implications for designing immunotherapies.

The previous study using ELISPOTs found that fewer eBL patients produce EBNA-1 specific IFN-γ compared to healthy controls [22]. ELISPOTs do not determine cell source of cytokine production therefore this assay could be measuring IFN-γ from cells other than T cells, such as Natural Killer cells. This may explain why we found no difference in the frequency of EBNA-1-specific IFN-γ T cell responses between patients and healthy controls, or by eBL outcome, in either CD4^+^ or CD8^+^ T cells. However, the magnitude of EBV-specific CD8^+^ IFN-γ was lower in non-survivors compared to survivors, suggesting that non-survivors may have an ineffectual EBV-specific IFN-γ T cell response. Moreover, most of the EBV-specific IFN-γ in non-survivors was not generated from an effector or memory subset but instead by a naïve-like T cell subset, while the IFN-γ in survivors was produced by CD45RA^-^CCR7^-^ T_EM_ cells. Cytokine responses were low in general, introducing a possible source of error in the characterization of cytokine T cell sources. However, the IFN-γ T_EM_ defect in non-survivors was specific to EBNA-1 and not PfSEA-1, suggesting that although the overall frequencies of T_EM_ cells are the same between the different groups, T_EM_ cells in non-survivors appear less functional at mounting EBV-specific pro-inflammatory responses. Taken together, this suggests that heterogeneity in immunity to EBV within the context of malaria exposure and eBL pathogenesis should be taken into account when designing immunotherapies to improve eBL survival.

Along with lower EBNA-1-specific IFN-γ T cell responses, non-survivors produced more EBNA1-specific IL-10. This has implications for immune surveillance of this latency I viral protein characteristically expressed by eBL tumors [10]. In addition to EBNA-1-specific IL-10, non-survivors also produced more PfSEA-1-specific IL-10 than survivors. Frequent malaria exposure produces tolerance to the malaria parasites and a reduction in clinical symptoms. However, IL-10 levels alone have not been found to correlate with malaria exposure, and all of our study participants are from the same area of holoendemic malaria transmission, making it unlikely that the higher IL-10 responses are due to differential malaria-exposure between the groups. One limitation of this study was that there were insufficient cells to simultaneously test responses to other EBV lytic and latent antigens, and it is unknown whether non-survivors would produce higher IL-10 levels to other antigens, or whether this is specific to EBNA-1 and PfSEA-1 alone.

In addition to Foxp3^+^ Treg cells, we identified CD4^+^Foxp3^-^ cells as sources of EBNA-1-specific IL-10. We hypothesize that these are regulatory type 1 (Tr1) cells, a Foxp3^-^, IL-10 producing subtype of inducible Treg cells. Tr1 cells are not as well characterized as Foxp3^+^ Treg cells and have not received the same focus in tumor immunology research, but previous studies suggested that EBV, including other EBV^+^ tumors, induces Tr1 cells [7, 32]. The presence of Tr1 cells could indicate that Foxp3^+^ Treg cells are not the only cell type restricting eBL tumor response, which has implications for future Treg-modulating therapy in eBL.

This paper is the first association of Treg cells and survival in eBL. Ultimately, future work will aim to determine 1) whether Treg frequencies can be used as a predictive biomarker of relapse, and 2) whether Treg modulating therapy is a valid treatment option in eBL, given these children are still being exposed to natural malaria infections. Treg modulating drugs and checkpoint blockade inhibitors, which interfere with regulatory pathways, are an expanding field of new cancer therapeutics [33], that in general have not yet been applied in pediatric oncology. Low-dose intermittent cyclophosphamide (< 500mg/m^2^) has been shown to deplete Treg cells *in vivo*, and increases antigen-specific responses in therapeutic cancer vaccination [34, 35]. eBL patients in the present study are treated with high dose (1200 mg/m^2^) cyclophosphamide as part of their in-patient treatment regimen. During the course of this study some patients were inadvertently treated with cyclophosphamide doses that were greater or less than guideline dosages. Patients who received overdoses of cyclophosphamide had higher mortality rates compared to those receiving the appropriate dose, while those who were under-dosed had better survival rates [19]. Unfortunately not enough of these samples remained to accurately determine if the lower cyclophosphamide doses and enhanced survival was associated with lower Treg frequencies. All of the children included in this study received the same dosages of chemotherapy. If future studies indicate that Treg depletion enhances the anti-tumor immunity in eBL, chemotherapy regimens that use low dose cyclophosphamide could be interrogated to determine if this has a beneficial immune-modulatory effect.

Our current working model for eBL children who either do not survive or who relapse is one of a dysregulated equilibrium between EBV-specific immunity and tolerance. The induction of tolerance to EBV, a lifelong infection, may limit immunopathology but once a cell achieves oncogenic potential, the same regulatory response to EBV may inadvertently contribute to tumor tolerance. Future studies will aim to determine the possibility of establishing a clinically actionable Treg threshold that could function as an indicator of patient immune status and aid in clinical decision making, with the ultimate goal of increasing eBL survival.

## Supporting Information

S1 Fig Gating strategies. Gating strategy for identification of T cells from singlets, live CD3^+^ T cells (using ViViD live/dead exclusion dye with CD14 and CD19 dump channel), and CD4^+^ versus CD8^+^ cells (A). Within the CD4^+^ gate, identification of CD25^+^Foxp3^+^ Treg cells using CD25 and Foxp3 Fluorescence Minus One (FMO) stains (B). Identification of CD4^+^ IFN-γ^+^ and IL-10^+^ cells (C).
(TIF)

S2 Fig Absolute lymphocyte counts. Absolute lymphocyte counts from clinical laboratory obtained at the time of venous blood draw using a coulter counter. There were no statistically significant differences by Kruskal-Wallis test.
(TIF)

S3 Fig No differences with malaria parasitemia or tumor stage. (A) Among those that produced CD4+ IFN-γ in response to PfSEA-1 stimulation, there was no difference in the amount of IFN-γ production whether the patient had a concomitant malaria blood-stage infection or not. (B) Among those that produced CD4+ IL-10 in response to PfSEA-1 stimulation, there was no difference in the amount of IL-10 production whether a patient had a concomitant malaria blood-stage infection. (C) When stratifying eBL patients by St. Jude/Murphy tumor staging, there was no association with Treg frequencies (p = 0.5731 by Mann-Whitney).
(TIF)

S4 Fig Overall frequencies of CD45RA and CCR7 expressing T cell subsets do not differ between non-survivors, survivors, and healthy controls. (A) Gating strategy to identify CD8^+^ CD45RA and CCR7 subsets. Frequencies of CD8^+^ and CD4^+^ CD45RA^-^CCR7^+^, CD45RA^+^CCR7^+^, CD45RA^-^CCR7^-^, CD45RA^+^CCR7^-^ cells (B, C). ● Non-survivors; ■ Survivors; ▲Healthy controls
(TIF)

S1 Table No differences in EBNA-1-specific IFN-g responses between health controls and patients with eBL. (A) Number of CD4^+^ EBNA-1 specific IFN-γ responses among eBL patients and healthy controls (p = 0·2591, Fisher’s exact test). (B) Number of CD8^+^ EBNA-1 specific IFN-γ responses among eBL patients and healthy controls (p = 0·2719, Fisher’s exact test).
(DOCX)
