# Supplementary figures and images for: Regulatory T Cells in Endemic Burkitt Lymphoma Patients Are Associated with Poor Outcomes: A Prospective, Longitudinal Study
Source: PLoS One. 2016 Dec 29;11(12):e0167841. doi: 10.1371/journal.pone.0167841 (PMC5199096; doi:10.1371/journal.pone.0167841)

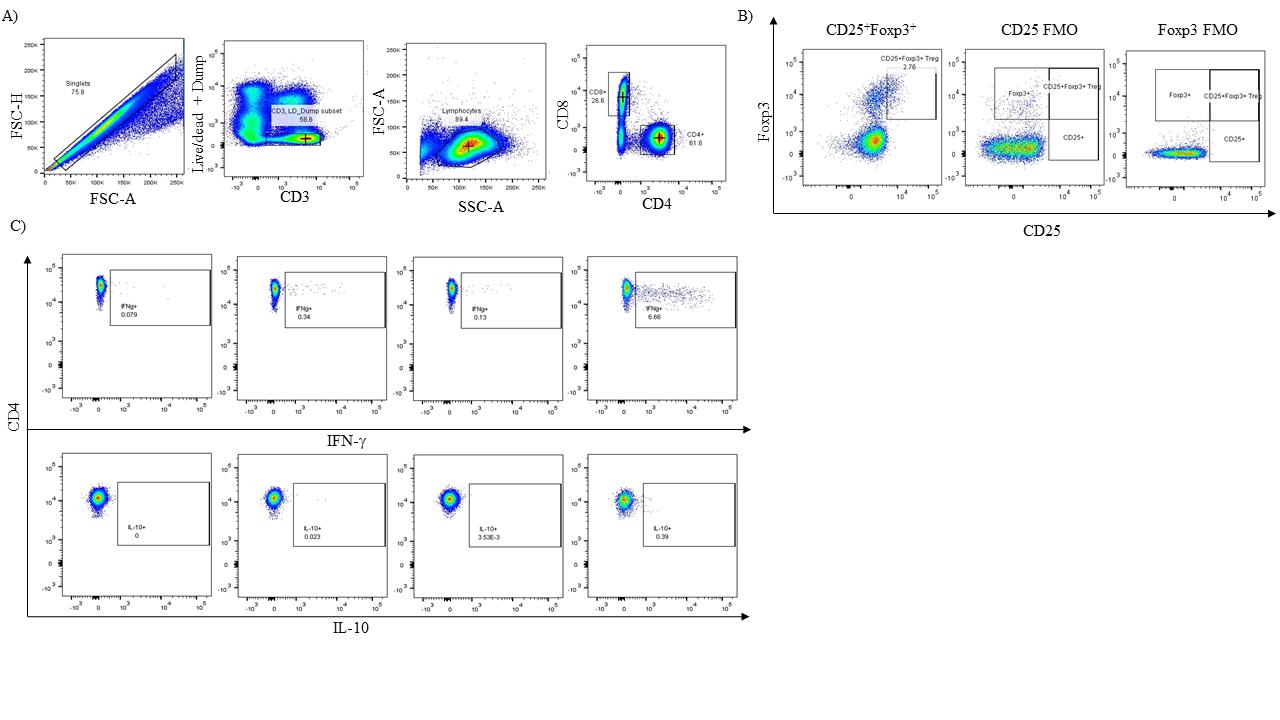

Supplement: S1 Fig — Gating strategy for identification of T cells from singlets, live CD3+ T cells (using ViViD live/dead exclusion dye with CD14 and CD19 dump channel), and CD4+ versus CD8+ cells (A). Within the CD4+ gate, identification of CD25+Foxp3+ Treg cells using CD25 and Foxp3 Fluorescence Minus One (FMO) stains (B). Identification of CD4+ IFN-γ+ and IL-10+ cells (C). (TIF) [file pone.0167841.s001.tif]

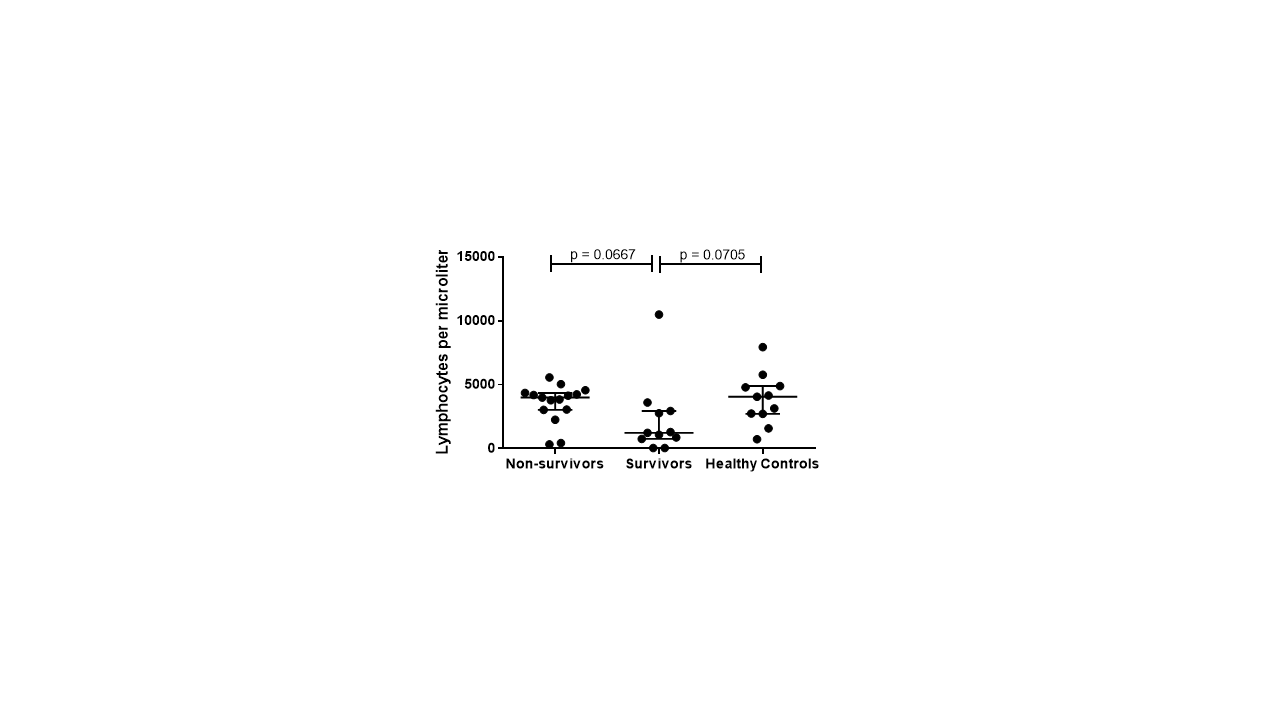

Supplement: S2 Fig — Absolute lymphocyte counts from clinical laboratory obtained at the time of venous blood draw using a coulter counter. There were no statistically significant differences by Kruskal-Wallis test. (TIF) [file pone.0167841.s002.tif]

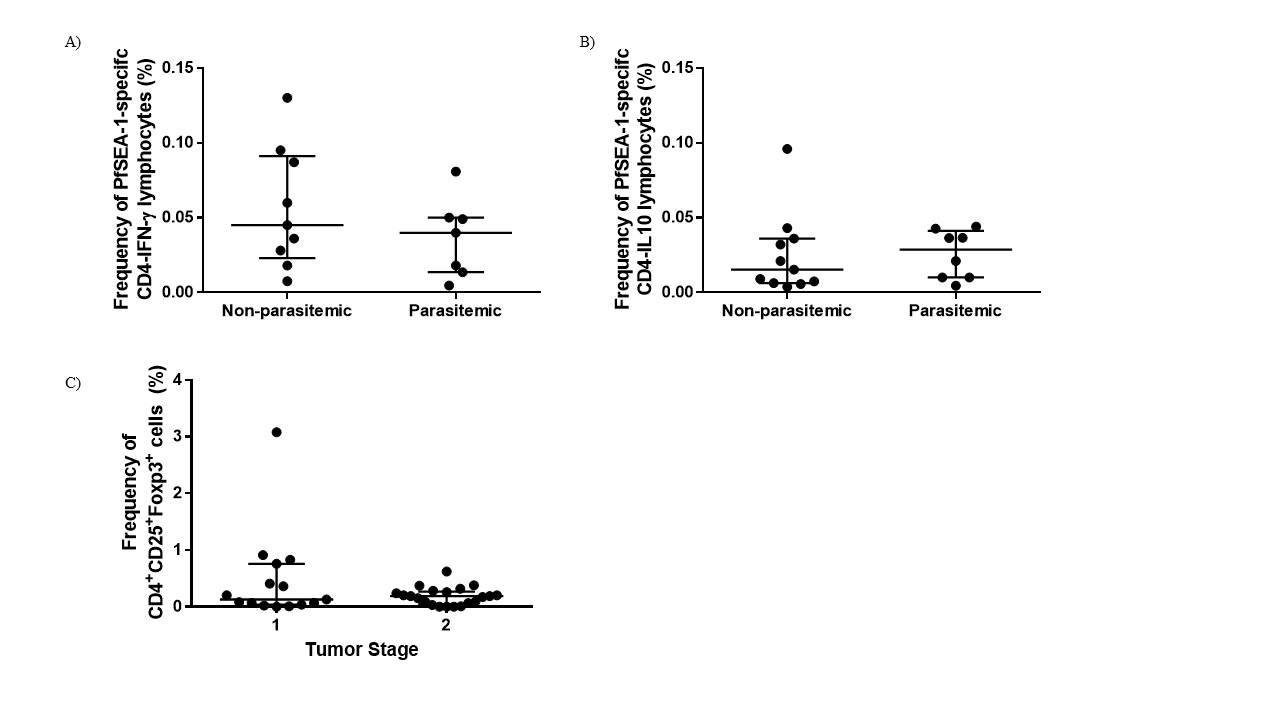

Supplement: S3 Fig — (A) Among those that produced CD4+ IFN-γ in response to PfSEA-1 stimulation, there was no difference in the amount of IFN-γ production whether the patient had a concomitant malaria blood-stage infection or not. (B) Among those that produced CD4+ IL-10 in response to PfSEA-1 stimulation, there was no difference in the amount of IL-10 production whether a patient had a concomitant malaria blood-stage infection. (C) When stratifying eBL patients by St. Jude/Murphy tumor staging, there was no association with Treg frequencies (p = 0.5731 by Mann-Whitney). (TIF) [file pone.0167841.s003.tif]

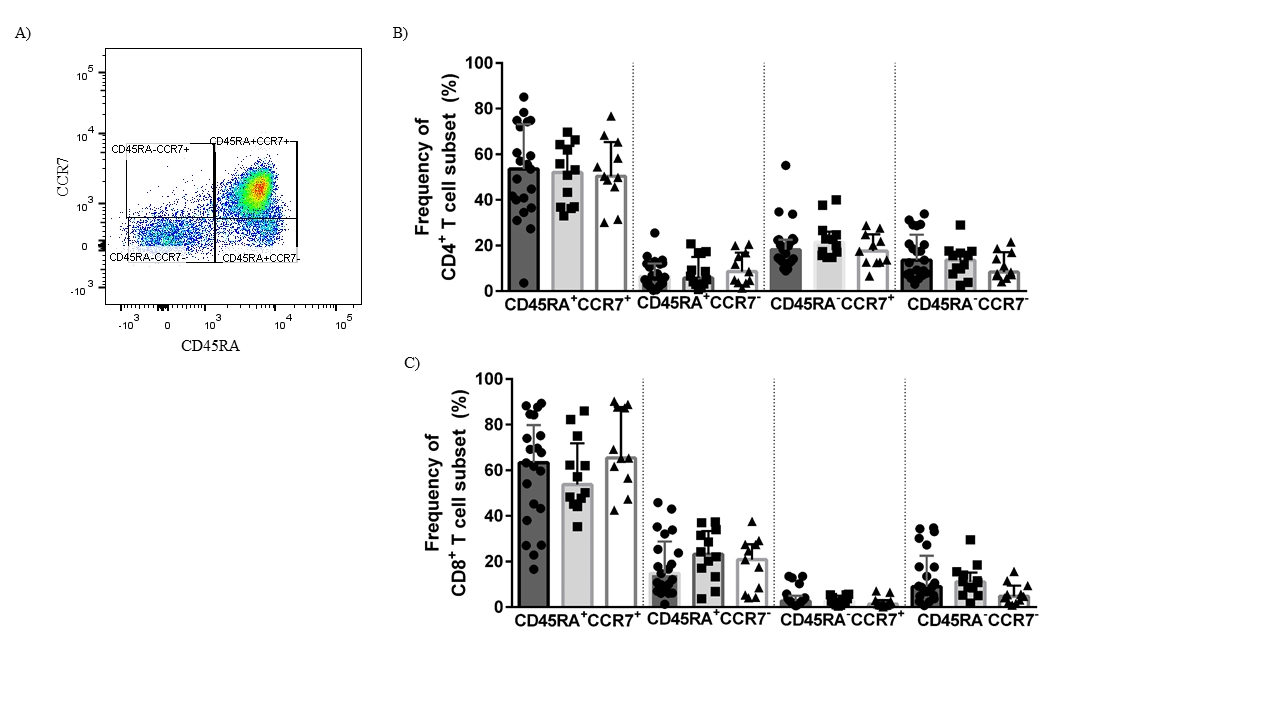

Supplement: S4 Fig — (A) Gating strategy to identify CD8+ CD45RA and CCR7 subsets. Frequencies of CD8+ and CD4+ CD45RA-CCR7+, CD45RA+CCR7+, CD45RA-CCR7-, CD45RA+CCR7- cells (B, C). ● Non-survivors; ■ Survivors; ▲Healthy controls (TIF) [file pone.0167841.s004.tif]
