# Supplementary material for: Regulatory T Cells in Endemic Burkitt Lymphoma Patients Are Associated with Poor Outcomes: A Prospective, Longitudinal Study
Source: PLoS One. 2016 Dec 29;11(12):e0167841. doi: 10.1371/journal.pone.0167841 (PMC5199096; doi:10.1371/journal.pone.0167841)
Supplement: S1 Table — (A) Number of CD4+ EBNA-1 specific IFN-γ responses among eBL patients and healthy controls (p = 0·2591, Fisher’s exact test). (B) Number of CD8+ EBNA-1 specific IFN-γ responses among eBL patients and healthy controls (p = 0·2719, Fisher’s exact test). (DOCX) [file pone.0167841.s005.docx]

**S1 Table.** **No differences in EBNA-1-specific IFN-g responses between health controls and patients with eBL.**

(A) Number of CD4^+^ EBNA-1 specific IFN-γ responses among eBL patients and healthy controls (p= 0·2591, Fisher’s exact test). (B) Number of CD8^+^ EBNA-1 specific IFN-γ responses among eBL patients and healthy controls (p= 0·2719, Fisher’s exact test).

**A)**

|  | **eBL patients** | **Healthy controls** |
| --- | --- | --- |
| IFN-γ EBNA-1 responder | 17 | 4 |
| IFN-γ EBNA-1 non-responder | 32 | 17 |

**B)**

|  | **eBL patients** | **Healthy controls** |
| --- | --- | --- |
| IFN-γ EBNA-1 responder | 16 | 6 |
| IFN-γ EBNA-1 non-responder | 20 | 17 |
